# Companion Tests and Personalized Cancer Therapy: Reaching a Glass Ceiling

**DOI:** 10.3390/ijms25189991

**Published:** 2024-09-17

**Authors:** Victoria Ferrari, Baharia Mograbi, Jocelyn Gal, Gérard Milano

**Affiliations:** 1 Department of Medical Oncology, Centre Antoine Lacassagne, University Côte d’Azur, 06189 Nice, France; 2 FHU OncoAge, IHU RespirERA, IRCAN, Inserm, University Côte d’Azur, CNRS 7284, U1081, 06000 Nice, France; 3 Epidemiology and Biostatistics Department, Centre Antoine Lacassagne, University Côte d’Azur, 06189 Nice, France; 4 Oncopharmacology Unit, Centre Antoine Lacassagne, University Côte d’Azur, 06189 Nice, France

**Keywords:** precision oncology, biomarker, lung cancer, artificial intelligence, companion tests, clinical trials

## Abstract

The use of companion diagnostics has become a standard in precision oncology in the context of ongoing therapeutic innovation. However, certain limitations make their application imperfect in current practice. This position paper underscores the need to broaden the notion of companion testing, considering the potential of emerging technologies, including computational biology, to overcome these limitations. This wave of progress should impact not only our representation of the analytical tool itself but also the nature of the tumoral sample under analysis (liquid biopsies). The complex inter-relationship between companion test guided-personalized therapy, and health agency policies for new drug agreements will also be discussed.

## 1. Introduction and Purpose of the Work

The purpose here is to describe the main applications of companion tests in the management of cancer treatment. A particular focus will be on determining epidermal growth factor receptor (EGFR) mutations in lung cancer, which currently serves as a concrete example of an optimized, targeted therapy approach in a specific tumoral localization.

Over the past two decades, cancer genomics has profoundly transformed our understanding of the natural history of the disease by uncovering crucial mechanisms of carcinogenesis [1]. This expanding knowledge of cancer pathways and the systematic application of advanced molecular biology techniques for diagnosis has fueled an explosive development of targeted therapies, ushering in a new era of precision oncology [2]. These breakthroughs have enabled earlier detection and more precise patient stratification by applying companion tests that guide personalized therapy.

Lung cancer, particularly non-small-cell lung cancer (NSCLC), perfectly illustrates the transformative power of precision oncology. The discovery of therapeutic targets and the subsequent development of companion test-based targeted therapy have opened a new paradigm in cancer medical treatment [3,4,5,6]. This evolution not only extends the benefits of precision oncology beyond mechanistic targets, but also significantly enhances our ability to manage lung cancer, improving patient outcomes.

The field of targeted drugs is continuously expanding, not just for lung cancer but also for colorectal cancer [7] and breast cancer [8]. Significant progress has been made with immune checkpoint protein inhibitors (ICIs) for advanced cancer patients, but a faithful companion test is still needed [9]. New targeting agents, including PARP inhibitors, proteolysis targeting chimeras (PROTACS), and molecular glues, are especially promising and still require companion test applications to ensure optimal personalization of treatment [10,11,12].

This position article is not intended to comprehensively review the vast field of personalized therapy associated with companion tests. The review of Valla and colleagues [13] has covered the whole area of companion biomarkers associated with personalized cancer therapy. The aim here is to highlight some key limitations in the accurate clinical use of these tests for major cancers, where targeting therapy is crucial. This article will pay particular attention to lung cancer, but typical examples from colorectal cancer and breast cancer will also be covered [14]. One of the main concerns raised by this position paper is the relative reliability of companion tests with a specific focus on the concrete case for PD-L1 expression guiding the application of ICIs and the example of BRAF mutation status for using BRAF inhibitors. This brief review will also emphasize the need to broaden the notion of companion testing, considering emerging technologies impacting the analytical tool and the nature of the tumoral sample under analysis. The complex intertwining of companion test guided-personalized therapy and health agencies’ policy for new drug agreements will also be discussed.

## 2. Companion Test-Current Status and Limits

### 2.1. Current Status

**Figure 1:**
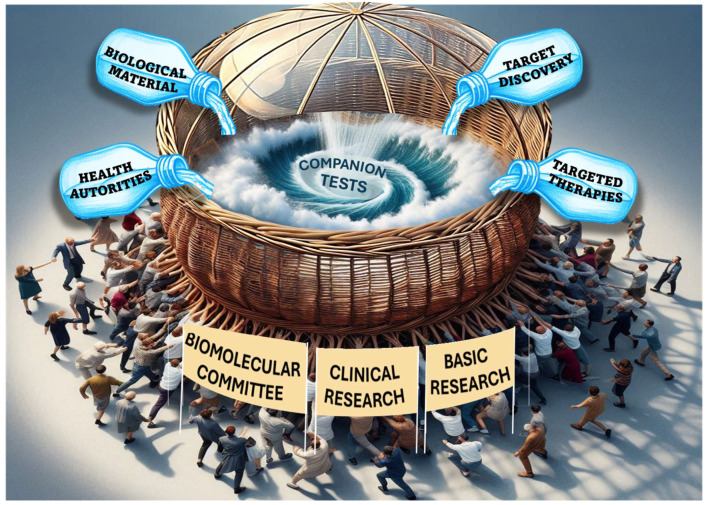
Companion tests and personalized cancer therapy: reaching a glass ceiling. Legend to figure: The ongoing global effort to develop new companion tests is crucial for advancing personalized cancer treatment. This wave of innovations is fueled by combined advancements in patient treatment (biomolecular committees), positive clinical research results, and continuous progress in basic research. New companion tests are tailored to align with emerging targeted therapies and treatment discovery. Adopting these tests largely depends on the biological material used for these tests and is subject to authorization decisions from health authorities. Nevertheless, this position article highlights some practical and theoretical limitations in the application of companion tests, symbolizing a glass ceiling (the words indicated in bold are used in the figure drawing).

This section will depict that the constant evolution in the duality between a given drug target and the specifically applied therapy has led to a complete paradigm shift in the standard of care for many tumor localizations, leading to personalized therapy [2] (Figure 1). In NSCLC notably, the move towards an expanded precision medicine based on individual tumor characteristics is well illustrated by the successive development of tyrosine kinase inhibitors targeting EGFR mutations, with trials comparing chemotherapy and targeted therapies systematically showing a clear superiority of targeted treatment. This success is primarily due to the application of companion tests, which play a crucial role in guiding these therapies [15,16]. The potential application use of companion tests has even gone beyond theragnostic interest. For instance, the emergence of resistance mechanisms in patients on anti-EGFR therapy may enlarge the notion of a unique predictive companion test [17]. Logically, it is now recommended to re-biopsy patients whose cancer continues to progress under EGFR-targeted tyrosine kinases to identify potential resistance mechanisms, which may themselves be the subject of a further application of targeted therapy [18].

Mutant RAS alleles play a central role in oncogenesis. RAS proteins remain essentially “undruggable”, and it took more than three decades before anti-RAS active drugs were designed [19,20]. Several clinical trials centered on KRAS mutation identification and targeting have been developed with marked therapeutic advances gained notably in NSCLC [20,21,22]. What is currently being observed in NSCLC is heralding a new therapeutic future in other major cancer localizations. Significant advances in breast cancer management have thus occurred and concern targeted therapy with immunotherapy by ICIs and the use of CDK 4/6 inhibitors, PARP inhibitors, and PI3K/AKT/mTOR inhibitors [23]. However, the broader implementation of companion testing, especially for the optimal use of CDK4/6 inhibitors in breast cancer, still requires robust validation [24].

### 2.2. Limits

This section will explain why some commonly used companion tests, such as PD-L1 tumoral expression, are not reliable predictors of therapeutic efficacy for immune checkpoint-based immunotherapy. This is mainly due to the complex intracellular regulation of PD-L1 expression. The section will also highlight the quantitative and qualitative limitations of currently used analytical methods like immunohistochemistry.

One of the limits of personalized therapy based on companion tests can be illustrated in the case of PD-L1. From a pathophysiological perspective, immune checkpoint molecules (PD-1 on T lymphocytes and PD-L1 on tumor cells) release cellular signals (immunological checkpoints) that inhibit T lymphocyte proliferation and anti-tumor activity. Blocking these checkpoints may restore the cytotoxic effect of the immune system on tumor cells [25,26]. Determining PD-L1 status by immunohistochemistry constitutes a basis for orienting immunotherapy by ICIs, extending the relevance of treatment into the perioperative setting of NSCLC [27]. There is, however, an inherent problem with tumor expression of PD-L1, which may be due to the complexity of PD-L1 regulation. PD-L1 expression is stimulated by interferon signaling controlled by the intra-tumoral presence of CD8+ T cells. On the other hand, PD-L1 expression is also regulated by intrinsic tumorigenic factors, which are independent of the immunological pathways [28,29]. Therefore, PD-L1 per se cannot provide sufficiently reliable intrinsic decision-making for applying anti-PD-1 therapy since the modulation of its expression may be independent of the immunological cellular context [30]. Consequently, identifying cellular and molecular features able to guide therapy by ICIs is challenging [31]. Analytical procedures based on multi-omics analysis could be a valuable avenue to explore to achieve optimal personalized therapy by ICIs [32], and hypothesis-generating biomarker approaches are being investigated in this sense [33]. In this quest for tumoral predictors of response to ICIs, tumor microsatellite instability (MSI) status offers a possibility. MSI reflects the tumor’s ability to generate neo-antigens; this tumor characteristic has been considered a valuable companion test for ICIs [34,35]. However, it remains counterintuitively possible to observe tumor sensitivity to an ICI-based therapy even in the presence of microsatellite-stable tumors. As an illustration, the report by Wang and coworkers on a phase II controlled trial in advanced colorectal cancer shows that the combination of an anti PD1 with an anti-VEGF and a HDAC inhibitor reaches an objective response rate of 44% in microsatellite-stable tumors which should be paradoxically refractory to ICIs [36]. This clinical finding was sustained and illustrated by pathological proof of a significant immune infiltrate generated by the presence of the two drugs combined with the ICI [36].

Another concern with companion test applications may spring from cellular mechanisms modulating the effect of the targeted agent against the impacted cellular pathway. This is the case with the BRAF V600E mutation and its therapeutic targeting by BRAF inhibitors in colorectal cancer, melanoma, and non-small cell lung cancer [37]. This key tumorigenic mutation has generated active clinical development with an extensive series of BRAF V600E inhibitors, whose optimal personalized application requires the tumoral presence of this mutation. However, a deleterious compensatory EGFR signaling consecutive to BRAF inhibition may complicate the use of BRAF V600E inhibitors [38]. EGFR expression is absent in melanoma cells but present at variable levels in colorectal cells [39]. Consequently, the clinical use of BRAF V600E inhibitors in colorectal cancer necessitates the supplementary application of cetuximab, a monoclonal antibody targeting EGFR [33]. Thus, the identification of colorectal cancer patients who could draw the highest benefit from anti-BRAF V600E targeted therapy should ideally be based not only on the presence of the BRAF V600E mutation but also take account of EGFR tumoral expression [39] for a personalized supplementation of BRAF targeting with cetuximab.

Given the ever-expanding range of companion tests associated with activable molecular targets, an alternative to conventional cancer treatments might be envisioned. This alternative strategy is based on treating the molecular abnormality rather than the tumor-bearing organ. The FDA has approved six anticancer drugs for seven tissue-agnostic indications between 2017 and 2022, representing 9 to 15 different tumor types [40]. The concept of tumor-agnostic indications has been further strengthened [14] and guided by the notion of treating the cancer driver gene independently of the tumoral localization [41]. However, a recent study of 10 published NCI-MATCH protocols (435 patients) has challenged this tempting alternative of a tumor-agnostic indication, showing that sensitivity to targeted therapies can still vary significantly depending on tumor origin [42]. Phase I and II studies have shown that despite sustained anti-tumor activity, the objective response rate to KRAS G12C inhibition oscillates widely according to the primary tumor site, ranging from 30% in bronchial cancers to 7% in colorectal cancers [21,43,44]. On the other hand, the negative results of the strictly controlled prospective trial SAPHIR 2 performed in 1462 patients with advanced breast cancer echoes the general lack of positive trials in the field of molecular companion test-based targeted therapy, providing evidence that, at least in advanced stages of the disease, cancer driver-based targeted therapy may not be superior to conventional therapeutic strategies [41].

A particular issue to be considered is the quality of the analytical tool used to characterize the companion test. In this respect, the case of antibody drug conjugates (ADCs) is particularly relevant. ADCs represent a promising novel class of anticancer therapeutics with, notably, Trastuzumab Deruxtecan (TDXd) combining the HER2 targeting monoclonal antibody Herceptin with a topoisomerase I inhibitor particularly active in breast cancer [45]. In NSCLC, the therapeutic efficiency of TDXd has also been demonstrated, and it is independent of HER2 expression level or the presence or the absence of amplification [46]. This unexpected finding raises questions about HER2 expression determined by immunohistochemistry as a faithful companion test to apply TDXd [47]. Pitfalls of immunohistochemistry have been reported for its validity in establishing companion tests, and thus, comprehensive genomic testing may be needed concurrently [48]. In this particular case of ADCs, where the payload carries the drug activity, using a companion test would rather serve to identify a potential target for the drug instead of predicting therapeutic efficacy. This view springs from the fact that the successful application of ADCs may depend on the presence of a bystander effect, which can be related to the release of the cytotoxic payload in the tumor microenvironment, independently of the incorporation of ADC in the tumoral target [49,50].

## 3. New Opportunities

Here, we discuss future applications that have the potential to enhance the performance of companion test measurements. Among these, the emergence of liquid biopsies, particularly circulating tumoral DNA, stands out as an ideal biological material for the broader use of companion tests in conjunction with associated treatments. Artificial intelligence-based algorithms will offer a wider range of solutions for a generalized practice of companion test measurement by integrating multiplex molecular profiling. Among the difficulties in successfully applying companion test-based therapy is the more or less complicated access to tumor material to perform the molecular profiling and the companion test assessment [51]. Moreover, as they are often difficult to repeat, these tissue sampling approaches do not allow the follow-up of tumoral clonal evolution over time, the response to treatment, or the detection of recurrence at an early stage. When considering NSCLC patients, only 18% have an adequate tumor sample for complete tissue genotyping for the eight guideline-recommended genomic biomarkers [52]. As an answer to this tumor sampling issue, minimally invasive liquid biopsies offer a particularly valuable alternative with the analysis of circulating blood components, including circulating tumor DNA (ctDNA) and tumoral cells [51]. A concrete application of ctDNA analysis to identify patients likely to benefit from a given treatment can be found in the study by Ros and colleagues, which looked at colorectal cancer treated with BRAF combination therapies [53]. The validated therapeutic schedule for BRAF inhibitor application in colorectal cancer is based on combining the RAF inhibitor (RAFi) with cetuximab [54]. BRAF mutation status is obtained from the primary tumor sampling. In a clinical-biological study by Ros and colleagues [53], it appears that patients with a high frequency of the BRAF V600E allele determined on the ctDNA material had better overall survival with triplet therapy (RAFi, MEKinhibitor, cetuximab) as compared to the standard doublet therapy (RAFi, cetuximab). Thus, determining the frequency of the BRAF V600E allele from early access to a ctDNA sample might provide, in this latter specific context, a better-personalized treatment option than merely determining the BRAF mutation status in the tumor itself, and this to the benefit of better treatment efficacy. 

Conditional approaches now combine the targeting of multiple proteins with key functions in the complex regulation of cancer progression [55]. As they become a therapeutic reality, they may also introduce a real difficulty for the widespread application of companion testing strategy, superseding the notion of a single companion test. Multiple comparison classifiers can now represent a higher level of complexity, and artificial intelligence-based algorithms are emerging that offer solutions by integrating multiplex molecular profiling [56,57,58]. Thus, complex patient-specific multi-omics signature could offer an alternative to the single companion test-based personalized therapy currently used. However, these approaches cannot be achieved without addressing the robustness of the analytical tools, which are necessary at both biological and technical levels. 

New targeted treatments based on bispecific antibodies, which are now in advanced stages of development in many tumor types, may also introduce an unavoidable level of complexity in companion test applications [59]. These antibodies, a logical extension of targeted therapies derived from companion diagnostics, are still in clinical trials. However, some, such as amivantamab for NSCLC with EGFR exon 20 insertion, have already been approved [60]. Their mechanism of action relies mainly on targeting both the primary target and one of the key resistance pathways, such as the MET pathway for amivantamab [61,62]. Clearly, in this complex case, a dual determination of the relevant targets represents a real need for companion test optimization. 

Last but not least, there is an evolutionary implication for health authorities regarding new drug development in the context of companion test-based treatment. Following an initial lack of enthusiasm from health authorities [63], probably reflecting the lack of positive clinical trials testing a personalized therapeutic strategy based on tumor genomics compared to conventional treatments, there has been increased communication between health authorities like the FDA, and the sponsors, forging a relationship between stakeholders developing therapeutics and those developing the companion tests [64]. New FDA regulatory paradigms have thus resulted in an accelerated approval of certain drugs benefiting from companion tests, unfortunately often in the absence of survival data [65]. This relative absence of survival data through strictly controlled prospective trials for CT-guided targeted therapy [66] may become a real issue between different health agencies controlling decision-making concerning new drug approvals [67]. This context also led to the fact that physicians may not recommend companion test-directed targeted therapy with low levels of FDA guidance [68]. Finally, further efforts are still needed to set up prospective controlled trials to assess the advantages or disadvantages of companion test-based targeted therapy compared to established conventional protocols in specific therapeutic contexts [69].

## 4. Conclusions and Perspectives

In this section, particular attention will be paid to current advanced analytical methods, such as proteogenomics, to discover and explore new targets for cancer treatment. Applying causal machine learning will enlarge the scope of companion test applications for guiding therapeutic decisions by integrating clinical and patient-related data.

A constant wave of clinical benefits for cancer management comes from personalized therapy based on a companion test associated with a given targeted treatment. As a glass ceiling limiting this expanding domain, a certain number of points need to be put into perspective regarding an optimal application of personalized therapy based on biological companion testing. In this respect, it is critical to develop standardized protocols for the use of companion diagnostics and strengthen collaboration between researchers and clinicians to optimize personalized treatment strategies [70]. The urgency of broadening the criteria for tumor dependency is clear, as this would include mutations that affect disease progression and assist in the optimal choice of targeted therapy in a “go-no-go” context. In breast cancer, for example, there is a clear need to develop a much more personalized therapy targeting CDK 4/6 than is currently available [71]. Although increasingly relevant in NSCLC, it is vital to broaden the development of targeted therapies and to expand the notion of companion diagnostic testing beyond the diseases we use as models today, namely those with EGFR mutations and ALK remodeling [72]. Multiplex testing, which allows for the simultaneous detection of multiple targetable biomarkers within a simple tissue sample (ideally a liquid biopsy), will undoubtedly continue to emerge in the near future. This strategy should benefit from applying AI-based algorithms to set up discriminative indexes for a “go-no-go” treatment approach [58]. Also particularly promising are the new advances in precision medicine, which come from applying functional drug screening to individual tumor samples to guide treatment decisions [55,73].

Further efforts are also needed to expand the scope of personalized medicine, as is currently being done to improve management of NSCLC [74,75]. There is also a clear need to identify tumoral targets for individualized treatment in new treatments, like PROTACS and next-generation degraders. Proteogenomics integrates mass spectrometry-based proteomics with genomics, providing a robust framework for discovering and exploring new targets for cancer treatment [76]. By integrating pan-cancer proteogenomic data from over 1000 patients across 10 cancer types, Savage and colleagues have unveiled a landscape of protein and peptide targets for drug repurposing and new targeted therapy applications [77]. Also, and perhaps most importantly, as recently pointed out, precision medicine increasingly requires broader partnerships and therapeutic modulation. Other optimal parameters of importance for therapeutic decisional strategy, in addition to conventional biological companion tests, should include clinical and patient-specific aspects across the cancer care continuum [78].
